# Evidence for the Role of Intracellular Water Lifetime as a Tumour Biomarker Obtained by In Vivo Field‐Cycling Relaxometry

**DOI:** 10.1002/anie.201713318

**Published:** 2018-04-14

**Authors:** Maria Rosaria Ruggiero, Simona Baroni, Stefania Pezzana, Gianni Ferrante, Simonetta Geninatti Crich, Silvio Aime

**Affiliations:** ^1^ Department Molecular Biotechnology and Health Sciences University of Torino via Nizza 52 Torino Italy; ^2^ Stelar Srl via E. Fermi 5 Mede (PV) Italy; ^3^ IBB-CNR via Nizza 52 Torino Italy

**Keywords:** intracellular water lifetime, magnetic resonance imaging, relaxometry, tumor detection

## Abstract

It was established through in vivo T_1_ measurements at low magnetic fields that tumour cells display proton T_1_ values that are markedly longer than those shown by healthy tissue. Moreover, it has been found that the elongation of T_1_ parallels the aggressiveness of the investigated tumour. The T_1_ lengthening is associated with an enhanced water exchange rate across the transcytolemmal membrane through an overexpression/upregulation of GLUT1 and Na^+^/K^+^ ATPase transporters. It follows that the intracellular water lifetime represents a hallmark of tumour cells that can be easily monitored by measuring T_1_ at different magnetic field strengths ranging from 0.2 to 200 mT.

Magnetic resonance imaging (MRI) has played a key role in the field of oncology over the last few decades. The prominent role of MRI relies on its superb spatial and temporal resolution, and its diagnostic power basically arises from the differences in the longitudinal (*T*_1_) and transverse (*T*_2_) proton relaxation times between healthy and pathological tissues. However, at the magnetic field strength of the currently available MRI scanners, changes in *T*_1_ do not appear to be sensitive enough to report on the particular aspects of the tumour stage.^1^ However, there is widespread opinion that at low magnetic field strength, the marked increase in *R*_1_ (=1/*T*_1_) observed in biological tissues might be beneficial towards improving the MRI diagnostic potential in tumour phenotyping.^2^

Herein, it is shown that the in vivo acquisition of 1/*T*_1_ nuclear magnetic resonance dispersion (NMRD) profiles (from 0.2 to 200 mT) fully supports this expectation as the observed *R*_1_ values at low magnetic fields (<0.2 T) enable the clear discrimination between tumours characterised by different metastatic potentials.

The 1/*T*_1_ NMRD profiles were acquired on fast field cycling (FFC) relaxometers, which are able to switch the magnetic field between different field strengths during the measurement procedure.^3^ A field cycle overcomes the problem of the low sensitivity at low fields and allows for the rapid acquisition of an NMRD profile (Figure 1 A). The most diffuse FFC relaxometers are designed for measurements of liquid or solid small samples (<1 cm^3^). To perform this study, prototype FFC‐NMR equipment was developed by STELAR (Mede, PV, Italy) for the acquisition of in vivo NMRD profiles on animal models. To host a mouse (ca. 20 g), a 0.5 T wide bore FFC magnet was used with the implementation of a dedicated 11 mm transmitter/receiver solenoid detection coil placed around the mouse's leg (Figure 1 B) where the tumour graft was located.

**Figure 1:**
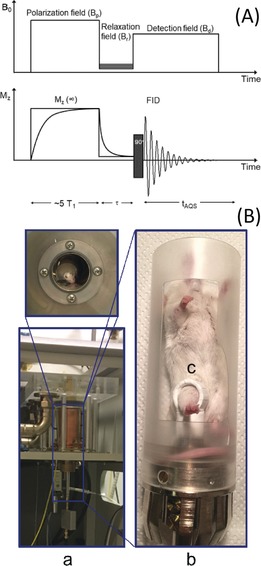
A) The FFC experiment: The nuclear spin polarization is built up during the pre‐polarization phase, at B_p_. Relaxation occurs during the evolution period (*τ*) at B_r_, then the NMR signal is detected at B_d_. The sequence is repeated, staggering *τ* each time. For B_r_>7 MHz, the cycle starts in the absence of any polarization field. B) Photographs of the FFC‐NMR relaxometer showing the introduced modifications for the in vivo acquisition: a) the FFC magnet; b) the mouse holding system; c) the transmitter/receiver coil around the mouse's leg.

In this study, mouse mammary adenocarcinoma cells, namely TS/A, 4T1, and 168FARN, were injected into the muscle of the hind limb to obtain tumour xenografts suitable for in vivo studies. The three cell lines were selected because they display different aggressiveness and metastatic potential (i.e., 168FARN<TS/A<4T1).^4^ When the tumour mass covers 65–85 % of the leg, the *T*_2_‐weighted images were acquired by MRI (1 T; Figure 2 A). The NMRD data points were obtained by using the procedure depicted in Figure 1 A (16 *τ* values).

**Figure 2:**
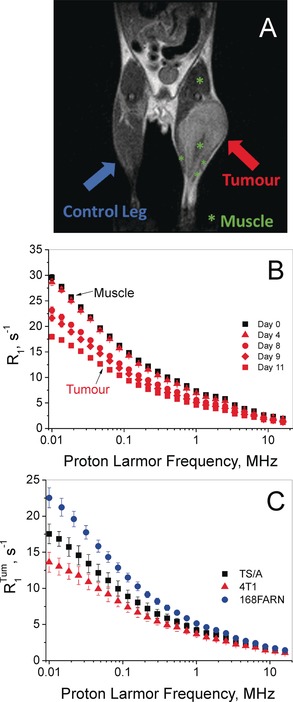
A) *T*_2_‐weighted MRI of the tumour‐bearing mouse (4T1 graft). B) 1/*T*_1_ NMRD profile of a mouse leg tissue before (day 0) and after the intramuscular injection of one million 4T1 cells. C) NMRD profiles of the tumour tissues grown on hind limbs: 4T1 (▴), TS/A (▪), and 168FARN (•) acquired 11±2, 13±3, and 25±1 days after intramuscular injection, respectively. *R*_1_^tum^ is the averaged relaxation rate normalized to the tumour mass fraction compared to the whole hind limb. Error bars report the standard deviation (SD).

The fitting of the magnetization decay curves (*M_z_*) for the determination of *T*_1_ was carried out by means of the monoexponential Bloch equation, despite the fact that the *M_z_* decay may display biexponential characteristics (see below). A simple inspection of the obtained profiles allowed us to clearly distinguish healthy from tumour tissue (Figure 2 B) as the tumour invariantly showed lower *R*_1_ values. Furthermore, the large differences observed in the *R*_1_ data at low fields provided insight into particular characteristics of the considered tumour grafts. The elongation of *T*_1_ followed the tumour size in different ways for the three models, essentially reflecting the differences in their aggressiveness (Figure 2 C). The observed behaviour clearly showed that the differences in *T*_1_ between the healthy and tumour tissue were significantly larger at low magnetic field strengths. The absence of extended necrotic/cystic areas was assessed by *T*_2_‐weighted image analysis (see the Supporting Information, Figure S1). As the averaged signal intensities measured on the three tumour models were not significantly different, it was possible to exclude that *T*_1_ elongation was mainly caused by the presence of microscopic necrotic areas.

To gain more insight into the factors determining the observed behaviour, one needs to remember that each *R*_1_ data point represents an average of the water *R*_1_ in different tissue microenvironments, basically 1) the extracellular (EX) space with an averaged *R*_1ex_ value and 2) the intracellular (IN) compartment with a more restricted water mobility, with a relaxation of *R*_1in_, with *V*_ex_ and *V*_in_ being the respective volume fractions. The intravascular volume may be neglected as it represents a tiny percentage of the total value.^5^ Water can cross the barriers between the two compartments, thus contributing to mixing, to some extent, towards the *R*_1_s of the IN and EX compartments. Therefore, *τ*_in_ and *τ*_ex_ (the IN and EX water residence times, respectively) have to be introduced in the model (Figure 3). Such exchange rates are correlated, according to the mass balance, through the volume fractions of the two compartments:

(1) $$\tau_{in}\times \;V_{ex}=\tau_{ex}\times V_{in}$$

**Figure 3:**
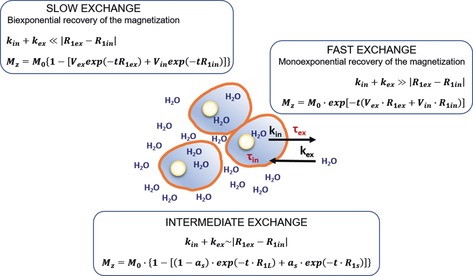
The water exchange regime and the resulting *M_Z_* value in a schematic representation of the relationship between the compartmentalized system formed by the IN and the EX space. In the case of intermediate exchange, *a_S_* and *R*_1S_ are the fraction and the rate constant for the apparent component with the shorter *T*_1_ (*R*_1S_=1/*T*_1S_); (1−*a*_S_)=*a*_L_ and *R*_1L_ are the fraction and rate constant for the apparent component with the longer *T*_1_ (*R*_1L_=1/*T*_1L_), and *t* is the running time for recovery by relaxation.

According to this bicompartmental model, the evolution time of *M_Z_* is dependent on the relationship between the absolute values of the “relaxation” term, |*R*_1in_−*R*_1ex_|, and an “exchange” term |*k*_in_+*k*_ex_| (where *k*_in_=1/*τ*_in_ and *k*_ex_=1/*τ*_ex_) in a relationship that has been previously defined as the NMR “shutter speed”.^6^

On the basis of this model (Figure 3), a monoexponential time course of *M_Z_* is expected only in a fast‐exchange regime, that is, when the condition |*k*_in_+*k*_ex_|≫|*R*_1in_−*R*_1ex_| is met. In this case, analysis of the saturation recovery (SR) data provides a single *R*_1_ value, which corresponds to the average between *R*_1in_ and *R*_1ex_ weighted by the volume fractions of the two sites. Conversely, when there is no exchange between the two compartments, the recovery of *M_Z_* will be biexponential, thus enabling the accurate determination of both *R*_1in_ and *R*_1ex_ values through a simple biexponential analysis of the SR data. In between, there is the intermediate‐exchange region in which the time evolution of *M_Z_* can still be biexponential, but the *R*_1s_ obtained from the fitting of the SR data can be “contaminated” by the exchange occurring between the two compartments.

The greater the exchange rate between the two compartments, the larger the *T*_1_ contamination arising by the EX compartment (endowed with a lower volume fraction). To estimate the different parameters, the *M_Z_* recovery was then re‐acquired over an extended number of *τ* intervals (*n*=48), to improve the sampling of both fast and slow *T*_1_ components. Moreover, an experimental approach to assess *R*_1ex_ values was pursued by measuring the 1/*T*_1_ profile of Matrigel (Figure 4).

**Figure 4:**
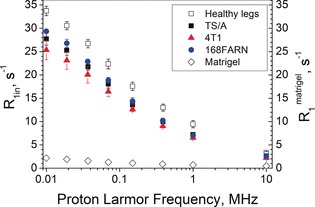
*R*_1in_ of mouse leg tissue: healthy legs and tumour‐bearing legs. Values were obtained from the NMRD data by fitting to the 2SX model. For comparison, the *R*_1_ values measured for Matrigel are reported (◊). Error bars indicate SDs from at least five independent experiments.

Matrigel is a gelatinous protein mixture secreted by Engelbreth–Holm–Swarm mouse sarcoma cells. It is a model of the EX environment found in many tissues and used as a substrate for cell culturing.^7^ The similarity of the NMRD profiles acquired on matrigel incubated for 72 h (Figure S2) in the absence or in the presence of 4T1, TS/A, and 168FARN cells supports the view that the presence of factors secreted by cells (e.g., proteins, enzymes, metabolites) does not affect the observed water proton *R*_1_s. This finding made us confident of the use of the Matrigel model to mimic the EX matrix compartment. By introducing the *T*_1_ values obtained for the Matrigel solution as the “long” *T*_1_ component, a good fit of the *M_z_* recovery curves was obtained using the mode equation for two‐site exchange (2SX model; see Section V in the Supporting Information). The *V*_ex_ was allowed to vary within a feasible range, in accordance with results already reported in the literature (0.09–0.19 for a healthy mouse hind limb, 0.15–0.5 for a tumourous mouse hind limb).6b, 8 The values of *τ*_in_ and *V*_ex_ given by the fitting are listed in Table 1.

**Table 1:** List of parameters derived by fitting the *M_z_* recovery data.

	In vivo experiment (NMRD profile)^[a]^		In vitro experiment (cell pellet)^[b]^
	*V*_ex_	*τ*_in_ [s]	*τ*_in_ [s]
muscle leg	0.14±0.02	1.24±0.25	–
4T1	0.22±0.08	0.68±0.20	0.023±0.009
TS/A	0.20±0.02	0.99±0.19	0.039±0.012
168FARN	0.15±0.01	1.12±0.32	0.111±0.014

[a] Data acquired on healthy and tumour‐bearing mouse legs between 0.01 and 10 MHz (number of *B*_r_: 8) were simultaneously fitted; the mean±SD is calculated from at least five independent experiments. [b] Data acquired on cells in the presence of Gd‐HPDO3A; the mean±SD is calculated from at least 4 independent experiments.

The most striking result from the fitting procedure is that the decrease in *τ*_in_ to indicate that the water exchange rate across the plasmalemmal membrane is a distinctive hallmark that differentiates between muscle (representative of healthy cells) and tumour cells. This finding clearly reports on the peculiar characteristics of the given tumour cell type. In fact, the IN water lifetime *τ*_in_ values reported in Table 1 for three breast cancer cell lines are inversely proportional to their metastatic potential. 4T1 cells are highly metastatic and form metastases in many organs (lungs, lymph nodes, brain, bone),4a TS/A cells display limited metastatic activity in the lungs,4a, 9 and 168FARN cells only produce local micro‐metastases.^9^

Support for this conclusion was gained by measuring *τ*_in_ values of the different cell lines in vitro, following a well‐established relaxometric procedure.^10^ For this purpose, measurements were carried out at 0.5 T in the presence of increasing amounts of the paramagnetic Gd‐HPDO3A complex in the EX space of cell suspensions. Then, the inversion recovery data were analysed according to the 2SX model.6b The obtained *τ*_in_ values are reported in Table 1. Although the drastically different experimental conditions caused a large decrease for the *τ*_in_ values obtained in vitro, they maintained analogous differences among the cell lines as observed in vivo, suggesting common determinants for *τ*_in_/*τ*_ex_.

Finally, it is worth noting that the IN relaxation times (*R*_1in_) appear rather insensitive to the cell type characteristics (Figure 4). These results confirm that the *R*_1_ values of IN water are markedly higher than those of EX water. IN water is embedded in an organized and immobilized protein network (cytoskeleton) that can be considered as a dynamic gel, and is more ordered than EX water.^11^ Figure 4 clearly indicates that IN water is the principal component of the typical 1/*T*_1_ NMRD tissue profile whereas EX water has a higher mobility and a lower *R*_1_. The latter result, well understandable for vascular water, may appear a bit odd for the EX matrix, which contains a high protein concentration. However, the experimental finding of low Matrigel *R*_1_ values confirms the hypothesis that this low value is most likely due to the higher water mobility of these proteins.

It follows that the main determinant of the elongation of *T*_1_ in tumour cells is *τ*_in_, which decreases as the aggressiveness increases.

Examples of 1/*T*_1_ NMRD profiles acquired on fresh or thawed surgical specimens have been reported previously.^12^ However, the use of ex vivo samples has the flaw that it cannot take into account the dynamics of water mobility, which is a key term determining the NMRD profile.

Why do tumour cells display a shorter *τ*_in_ value than healthy cells? The answer may rely on the increased glycolytic activity of tumour cells, which leads to an increased production of metabolites with a consequent increase in the IN osmotic pressure. The tumour cells cope with this issue by increasing the exchange rate of water with the external compartment. Therefore, to gain further support for our views, the expression of glucose transporter GLUT‐1 and Na^+^/K^+^ ATPase was evaluated by immunofluorescence (Figure 5).

**Figure 5:**
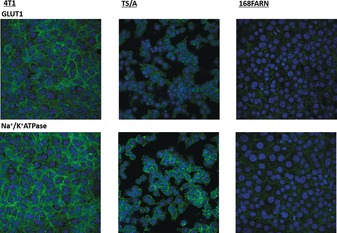
Immunofluorescence confocal images (63X). Cells were stained for GLUT‐1 (upper panels) and Na^+^/K^+^ ATPase (bottom). Nuclei were counterstained with DAPI (blue).

To unambiguously demonstrate that the expression of these transporters is directly correlated with the decrease in *τ*_in_ observed for the three cell lines (4T1<TS/A<168FARN), the effect of the inhibition of GLUT‐1 and Na^+^/K^+^ ATPase was assessed. Cells were incubated for 24 h in the presence of 5 μm of WZB117,^13^ the GLUT‐1 inhibitor, or 100 μm of Ouabain,^14^ the Na^+^/K^+^ ATPase inhibitor. Then, *τ*_in_ was determined on the cell lines “in vitro” following the above described procedure. Cell vitality, determined by MTT assays, was >90 % also after WZB117 and Ouabain treatment (see Figures S9 and S10). The treated 4T1 and TS/A cells showed a marked increase in *τ*_in_ (Figure 6), confirming the suggestion about the relevant role of the expression of this transporter in the modulation of transcytolemmal water exchange rates. As expected, the longer *τ*_in_ of 168FARN is less dependent on both inhibitors.

**Figure 6:**
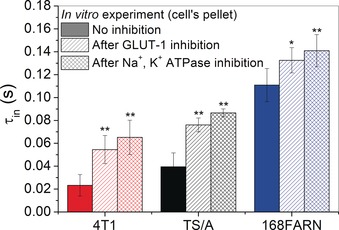
*τ*_in_ values determined “in vitro” on cells with and without GLUT‐1 and Na^+^/K^+^ ATPase inhibition. Errors bars indicate SDs from at least four independent experiments. *P<0.05; **P<0.01; Student's t‐test.

The results reported herein show that 1/*T*_1_ NMRD profiles measured in vivo on implanted mammary tumours clearly allow for the assessment of marked *T*_1_ increases, with respect to healthy tissues, that occur at low magnetic fields (<0.2 T). This achievement may open new horizons for the non‐invasive evaluation of tumour metabolic phenotypes by providing useful and more detailed information related to the metastatic propensity of the tumour without requiring the administration of exogenous contrast agents. This finding outlines the dependence of the observed *T*_1_ on the transcytolemmal water exchange rate when the two involved compartments have a sufficiently different *R*_1_, as observed at low magnetic fields (<0.2 T). The simultaneous fitting of the *M_z_* over an extended range of magnetic field strengths allows for a good estimation of *τ*_in_. Water transport across the plasma membrane is crucial to cell function. Cell water content and cell volume are related to the concentration of IN osmotically active compounds as well as to the EX tonicity. Cations, anions, and other metabolites are transported across the cell membrane by active transporters whose up/downregulation occurring in the presence of a pathological state can act as a specific reporter of the cellular state. *τ*_in_ reports on the activities of a number of transporters, and collectively, it may represent a hallmark of tumour‐cell aggressiveness. The *τ*_in_ is the result of contributions from a number of sources, including overexpression/upregulation of transporters such as GLUT‐1 and Na^+^/K^+^ ATPase. We may conclude that the measurement of transmembrane permeability provides insight for more specific assessments of the pathophysiological status of tumours. Even though FFC‐NMR instrumentation is not endowed with spatial resolution, the fundamental knowledge obtained in this study can enable new diagnostic opportunities in oncology that were previously unrecognized and are potentially transferable to the two prototype human‐whole‐body‐sized FFC‐MRI scanners recently built at Aberdeen University by Lurie and co‐workers. Pilot studies performed on these FFC‐MRI scanners have already demonstrated the potential use of FFC‐MRI in a range of several pathologies such us musculoskeletal and cardiovascular diseases.^15^

## Conflict of interest

The authors declare no conflict of interest.

## Supporting information

As a service to our authors and readers, this journal provides supporting information supplied by the authors. Such materials are peer reviewed and may be re‐organized for online delivery, but are not copy‐edited or typeset. Technical support issues arising from supporting information (other than missing files) should be addressed to the authors.

Supplementary
