# Supplementary material for: Evidence for the Role of Intracellular Water Lifetime as a Tumour Biomarker Obtained by In Vivo Field‐Cycling Relaxometry
Source: Angew Chem Int Ed Engl. 2018 Apr 14;57(25):7468–72. doi: 10.1002/anie.201713318 (PMC6175164; doi:10.1002/anie.201713318)
Supplement: Supplementary file 1 — Supplementary [file ANIE-57-7468-s001.pdf]

Angewandte Chemie
Eine Zeitschrift der Gesellschaft Deutscher Chemiker

Supporting Information

Evidence for the Role of Intracellular Water Lifetime as a Tumour Biomarker Obtained by In Vivo Field-Cycling Relaxometry

Maria Rosaria Ruggiero[+], Simona Baroni[+], Stefania Pezzana, Gianni Ferrante, Simonetta Geninatti Crich, and Silvio Aime*

anie_201713318_sm_miscellaneous_information.pdf

Table of Contents

I) Cell Cultures

II) Animal Models

III) Magnetic Resonance Imaging

IV) *In vitro* Nuclear Magnetic Resonance Dispersion profile (NMRD) of matrigel

V) *In vivo* NMRD profile

VI) *In vitro* determination of membrane permeability by relaxometric procedure

VII) Immunofluorescence of GLUT1 transporter and Na+/K+ ATPase

VIII)Inhibition of GLUT1 transporter and Na+/K+ATPase and determination of membrane permeability

IX) Theoretical Methods: NMRD data analysis

X) References

I) Cell Cultures.

4T1 (ATCC® CRL-2539™), TS/A and 168FARN cell lines were initially derived from a spontaneous breast tumour growing in a BALB/c mouse. 4T1 (purchased from American Type Culture Collection (ATCC, USA)) and TS/A (kindly provided by prof. F. Cavallo's group, University of Turin) were grown in RPMI 1640 medium supplemented with 10% fetal bovine serum (FBS), 100 U/mL Penicillin (P) with 100 µg/ml Streptomycin (S) and 4 mM Glutamine (Gln). 168FARN (kindly provided by prof. V. Poli's group, University of Turin) were grown in Dulbecco's modified Eagle's media containing 25 mM glucose (Glc), 6 mM Gln, 100 U/ml of P, 100 µg/ml of S, not-essential aminoacids 1 mM (NEAA) and 10% FBS. Cells were cultured in 5% CO_2 / 95% air at 37 °C in a humidified chamber and were split every 2 to 3 days. All cells were tested negative for mycoplasma by MycoAlert™ Mycoplasma Detection Kit. All materials were purchased from Lonza (Basel, Switzerland).

II) Animal Models.

6-old-week female BALB/c mice were inoculated in their muscle hind-limb with 1 million of 4T1 (group 1, n=5), TS/A (group 2, n=5) or 168FARN (group 3, n=7) cells suspended in 100 µl of Phosphate Buffered Saline (PBS). NMRD profiles were acquired 11(±2), 13(±3), 25(±1) days after cell inoculation for 4T1, TS/A and 168FARN, respectively. A fourth group of untreated mice (n=8) was used as control. BALB/c mice (Charles River Laboratories Italia S.r.l., Calco Italia) were maintained under specific pathogen free conditions in the animal facility of the Molecular Biotechnology Center, University of Turin, and treated in accordance with the EU (EU2010/63) and Italian (d.lgs 26/2014) regulations. Before being undergone imaging and nuclear magnetic resonance experiments, mice were anaesthetized with a mixture of tiletamine/zolazepam (Zoletil 100; Vibac, Milan, Italy) 20 mg/kg and xylazine (Rompun; Bayer, Milan, Italy) 5 mg/kg. When tumours reached 10 mm mean diameter, mice were euthanized for ethical reasons. The animal treatment protocol was approved by the Italian Ministry of Health (Authorization number 807/2017-PR).

III) Magnetic Resonance Imaging.

The T_1-weighted images were acquired at 1 T on Aspect M2-High Performance MRI System (Aspect Magnet Technologies Ltd., Netanya, Israel) consisting of a NdFeB magnet, equipped with a 35 mm solenoid Tx/Tr coil of inner diameter 35 mm. This system is equipped with fast gradient coils (gradient strength, 450 mT m^{-1} at 60 A; ramp time, 250 µs at 160 V) with a field homogeneity of 0.2−0.5G. The MR image was acquired with a Fast Spin Echo sequence (FSE) (TR 3000 ms; TE 50 ms; number of slices 11; slice thickness 1 mm; FOV 50x50 mm; matrix 168 × 160, Flip angle 90°). Tumour volume was determined using the ITK-SNAP software.

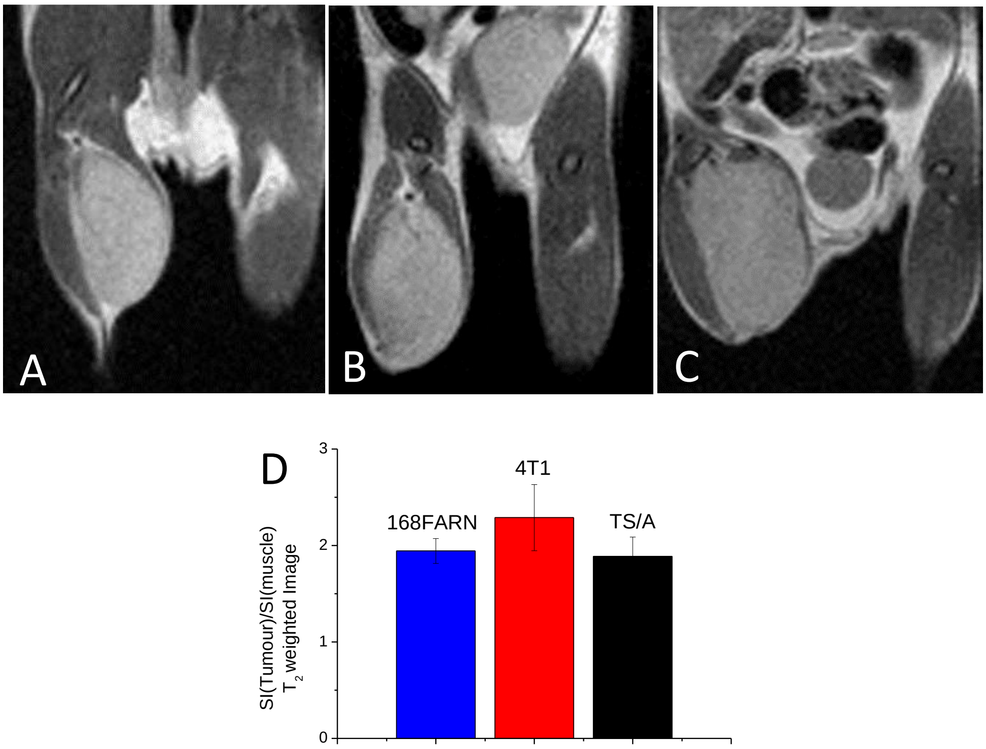

Figure S1. Three representative T_2-weighted images of tumours, acquired at 1 T (Aspect M2) 10, 13 and 25 days after intramuscular injection of 168FARN (A), 4T1 (B) and TS/A (C), respectively. Panel D shows the SI measured on tumour tissues normalized to healthy muscle SI. Experimental data are the averages of at least 5 animals per tumour type (± SD).

IV) *In vitro* Nuclear Magnetic Resonance Dispersion (NMRD) profiles of Matrigel

The protein concentration of Corning® Matrigel® matrix is 8-12mg/ml, approximately 60% laminin, 30% collagen IV, and 8% entactin as indicated by manufacturing company. Corning® Matrigel® matrix also contains heparan sulfate proteoglycan, TGF-β, epidermal growth factor, insulin like growth factor, fibroblast growth factor, tissue plasminogen activator. There is also residual matrix metalloproteinases derived from the tumor cells. The $1/T_1$ NMRD profiles of Matrigel were measured over a continuum of magnetic field strength from 0.00024 to 0.24 T (corresponding to 0.01–10 MHz proton Larmor frequency), on the fast field cycling (Stelar SMARTRACER). The relaxometer operates under complete computer control with an absolute uncertainty in the $1/T_1$ values of ±2%. The typical field sequences used were the NP sequence between 10 and 7 MHz and PP sequence between 7 and 0.01 MHz. The observation field was set at 7.2 MHz while the polarization field at 9 MHz. T_1 was determined by the saturation recovery method. 16 values of delay (τ) between pulses have been used. The number of averaged experiments was 2. The NMRD profile reported in Figure 4 of the main text was acquired at 25°C on 0.5 ml of gelled Corning Matrigel without any modification. NMRD profiles of different Matrigel samples were then acquired after growing tumour cells (4T1, TS/A and 168FARN) in this medium for 72 h. Briefly, cells were re-suspended in cold Matrigel at a density of $2.5x10^5$/ml, $2.5x10^5$/ml, $5x10^5$/ml of 4T1, TS/A and 168FARN cells, respectively and incubated at 37°C for 72h. Before the acquisition of the NMRD profiles matrigel was collected by cell plates by de-polymerization at 4°C and, after transferring in the glass NMR tube, it was re-polymerized at room temperature.

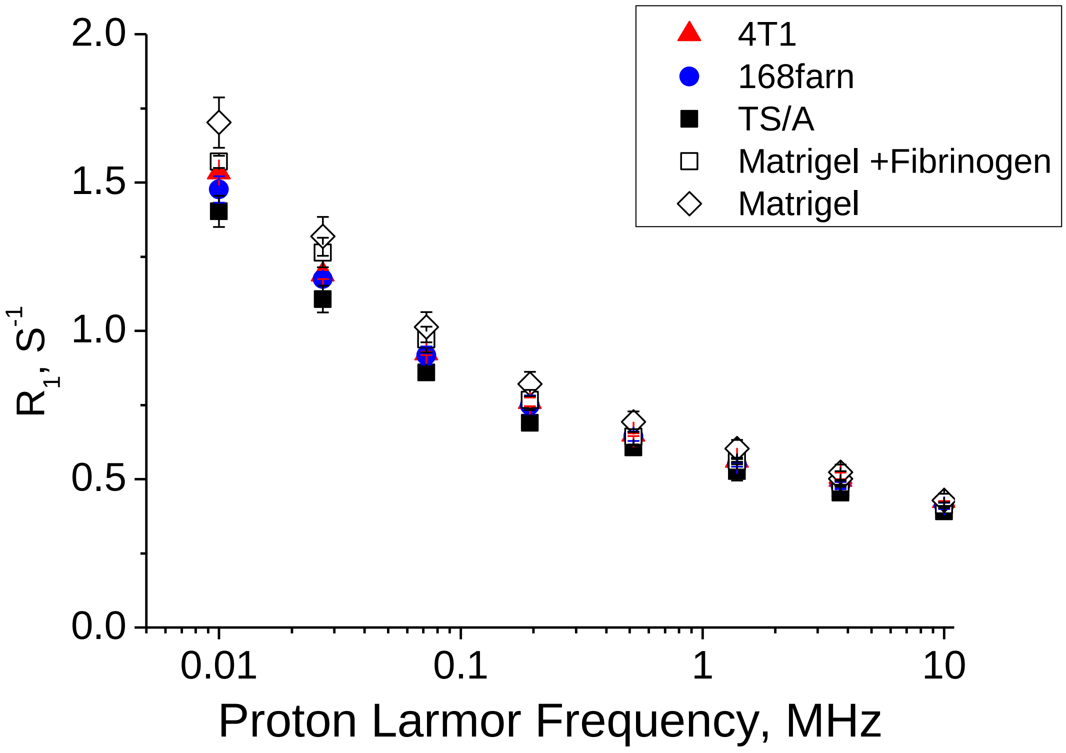

Figure S2. NMRD profiles acquired at 25°C on Matrigel samples incubated for 72 h at 37°C in the absence (open rhombus) or in the presence of 4T1 (red filled triangle), TS/A (black filled circle) and 168FARN (blue filled circle) cells or 2.5mg/ml of fibrinogen (open squares).

V) *In vivo* Nuclear Magnetic Resonance Dispersion profile (NMRD).

In vivo NMRD profiles were acquired over a continuum of magnetic field strength from 0.01 to 16 MHz Proton Larmor Frequencies on the Stelar SPINMASTER FFC-NMR relaxometer (Stelar S.n.c., Mede (PV), Italy) equipped with a 40 mm 0.5 T FC magnet and a dedicated 11 mm solenoid detection coil. The relaxometer operates under complete computer control with an absolute uncertainty in the $1/T_1$ value of ±2%. The typical field sequences used were Non-Polarized sequence (NP/S) between 16 and 7 MHz and the Pre-Polarized sequence (PP/S) between 7 and 0.01 MHz. The observation field was set at 14.5 MHz, while the polarization field at 13 MHz. For the acquisition of the NMRD profiles reported in Figure 2, sixteen delay (τ) values between pulses were used, with a switching time of 4 ms, and 1000 data points for each τ were acquired. T_1 was determined by the saturation recovery method and analysed as a mono-exponential decay (Bloch equation). In order to take into account the contribution of the remaining healthy muscle the relaxation rate of tumour tissues (R_1^{Tum}) were calculated according to the following formula:

$$R_1^{Tum} = \frac{R_1 - (1 - \mathrm{x})R_1^{mus}}{\mathrm{x}}$$

where R_1 is the observed relaxation rate arising from tumour bearing leg, x is tumour volume fraction and R_1^{mus} is the observed relaxation rate of the healthy hind-limb before tumour cell implantation.

Table S1 reports tumour volume fraction (x) of the different tumour bearing mice used in this study and the calculated R_1^{Tum} at 0.01 MHz with their standard deviations.

The NMRD profiles reported in Figure 4 and the parameters reported in Table 1 of the main manuscript were obtained re-acquiring the tumours relaxometric profiles using an increased τ number (n=48) over a longer time range, in order to sample better both fast and slow magnetization components. These profiles were acquired at eight different relaxation field strengths (0.01, 0.02, 0.037, 0.07, 0.15, 0.39, 1 and 10 MHz). Data were simultaneously analysed with Origin software (OriginPro 8.5.0 SR1, OriginLab, Northampton, MA, Levenberg-Marquardt algorithm), sharing the V_{ex} end τ_{ex} parameters maintaining R_{1ex} fixed to the Matrigel values obtained in a

separated experiment. (see above) The V_{ex} was allowed to vary within a feasible range, in accordance with results already reported in the literature (0.09-0.19 for healthy mouse hind-limb, 0.15-0.5 for tumour mouse hind-limb (see ref. 6b and 8 in the main text).

Table S1. Tumour volume fractions and relaxation rates at 0.01 MHz of the different tumour bearing mice models.

Mouse	Tumour volume fraction, x (SD)	R_1 (SD), s^{-1} 0.01 MHz	R_1^{mus} (SD), s^{-1} 0.01 MHz	R_1^{Tum} (SD), s^{-1} 0.01 MHz
4T1				
1	0.670 (0.112)	18.0 (0.5)	29.6 (0.6)	12.2 (0.6)
2	0.712 (0.014)	18.0 (0.4)	29.5 (0.5)	13.3 (0.5)
3	0.663 (0.026)	18.5 (0.4)	30.8 (0.7)	12.3 (0.7)
4	0.831 (0.019)	14.7 (0.4)	29.0 (0.5)	11.8 (0.5)
5	0.720 (0.023)	20.0 (0.5)	31.8 (0.5)	15.5 (0.5)
TS/A				
6	0.650 (0.049)	20.8 (0.5)	29.0 (0.5)	16.4 (0.5)
7	0.647 (0.028)	22.0 (0.5)	28.5 (0.6)	18.5 (0.6)
8	0.646 (0.058)	20.3 (0.5)	28.5 (0.5)	15.8 (0.5)
9	0.747 (0.006)	21.0 (0.5)	28.9 (0.5)	18.4 (0.5)
10	0.671 (0.014)	22.7 (0.4)	30.0 (0.5)	19.1 (0.5)
168FARN				
11	0.701 (0.064)	25.5 (0.4)	28.8 (0.5)	24.1 (0.6)
12	0.674 (0.035)	24.7 (0.4)	28.8 (0.5)	22.7 (0.6)
13	0.683 (0.044)	26.0 (0.4)	30.4 (0.5)	24.0 (0.5)
14	0.792 (0.024)	23.0 (0.5)	29.5 (0.5)	21.3 (0.5)
15	0.696 (0.013)	23.7 (0.3)	30.8 (0.5)	20.6 (0.5)
16	0.837 (0.005)	24.0 (0.4)	31.2 (0.5)	22.6 (0.5)

SUPPORTING INFORMATION

Figure S3 and S5 show representative fitting curves of both a hind limb muscle and a 4T1 tumour bearing mouse with their fitting residuals (Figure S4 and S6).

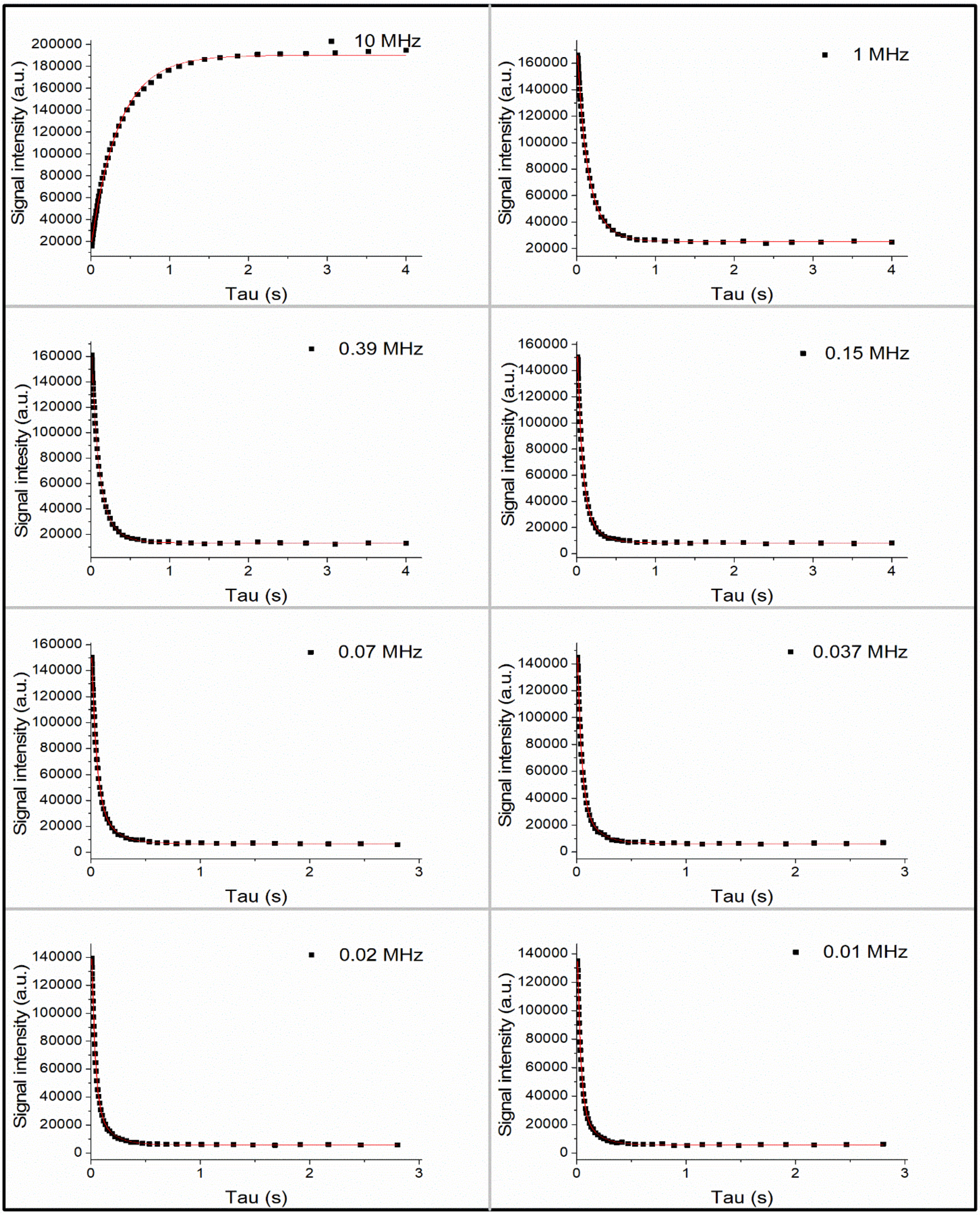

Figure S3. SR data from a hind limb muscle analyzed in terms of the 2SX model. Table S2 reports the parameter values obtained by the fitting.

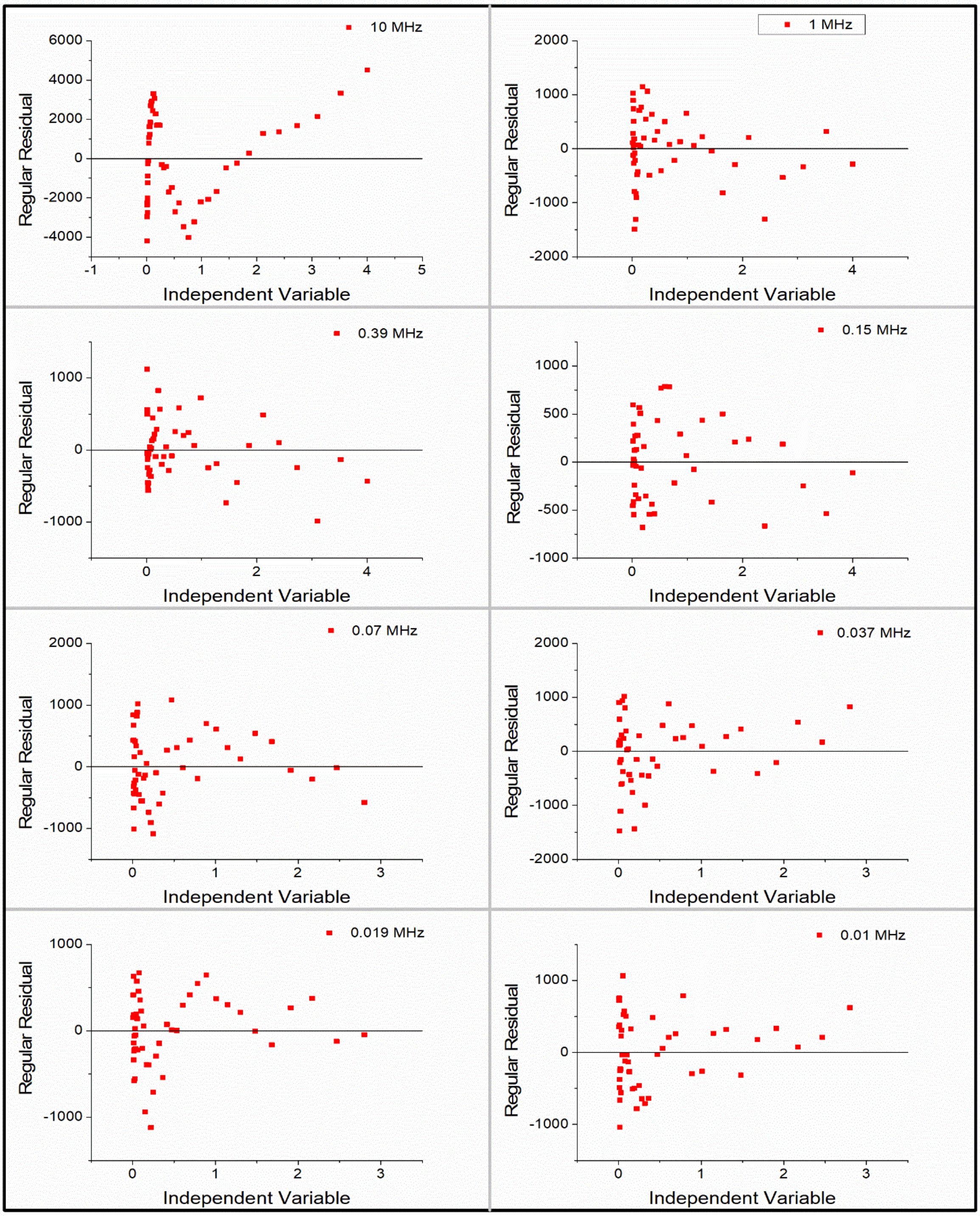

Figure S4. Residuals of the SR data fittings with the 2SX model reported in Figure S3.

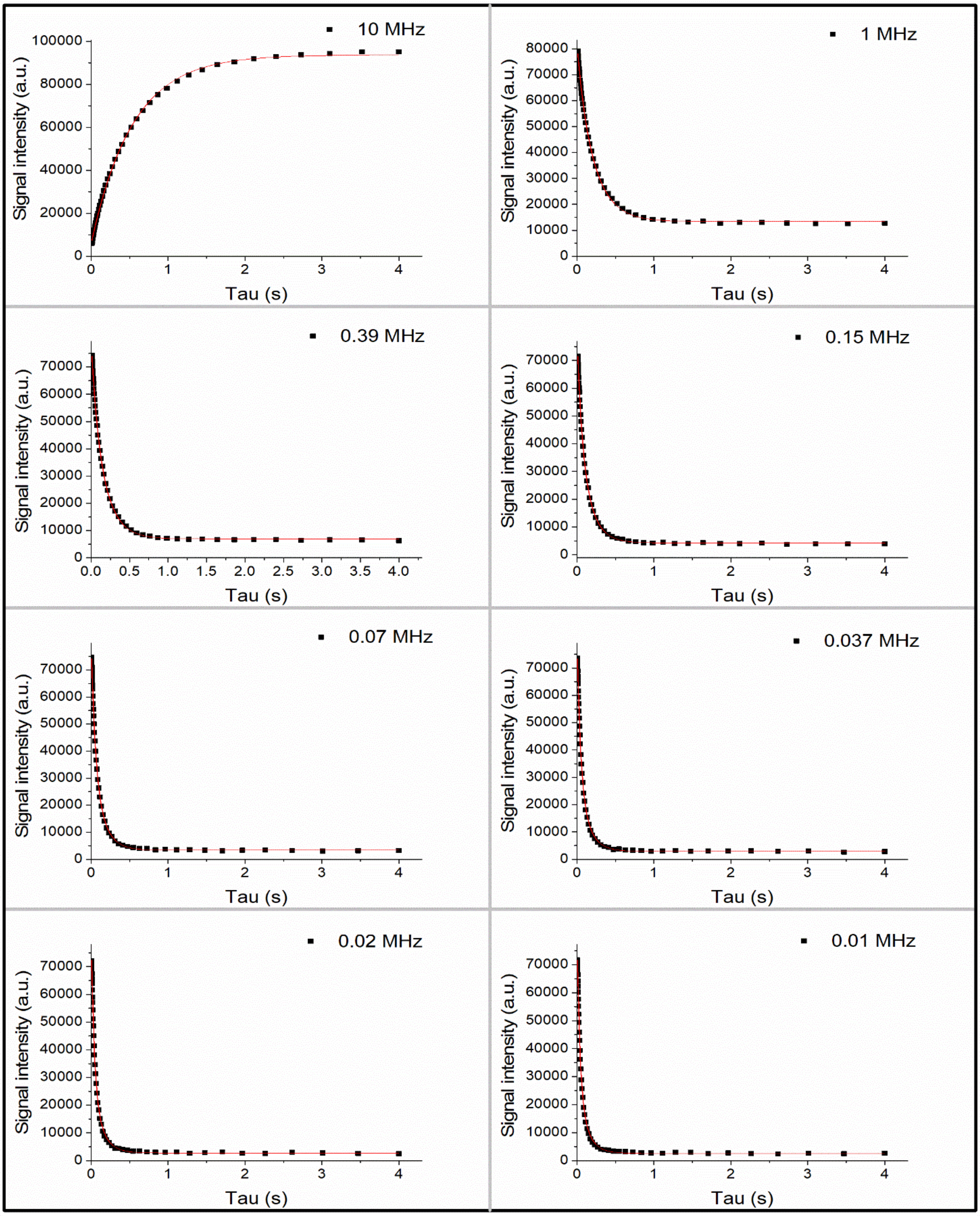

Figure S5. SR data from a 4T1 tumour bearing mouse leg, analyzed in terms of the 2SX model. Table S2 reports the parameter values obtained by the fitting.

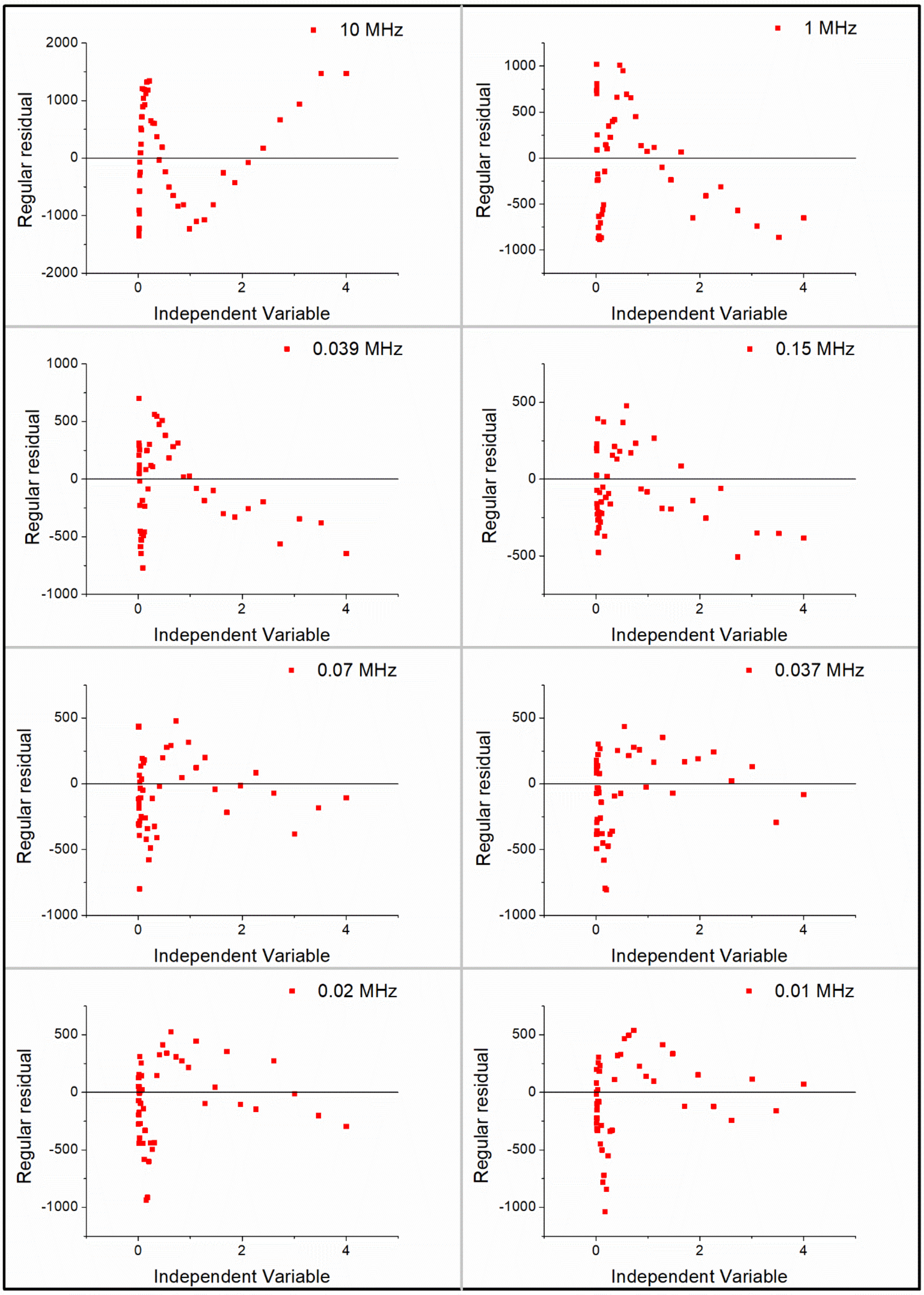

Figure S6. Residuals of the SR data fittings with the 2SX model reported in Figure S5.

Table S2. Parameters obtained from the 2SX model fittings of mice data reported in Figure S3 and S5.

Sample	Extracellular lifitime, τ_{ex} (s^{-1})[a]	Extracellular water mole fraction, V_{ex}[a]	Adj. R^2
Mouse leg	0.185 ± 0.012	0.134 ± 0.005	0.99971
4T1	0.145 ± 0.010	0.190 ± 0.010	0.99969

[a] The Standard Error is reported.

Figure S7 and S8: Mono-exponential fit of SR data measured at 0.01 and 10 MHz on a healthy and tumour mouse hind limb.

A

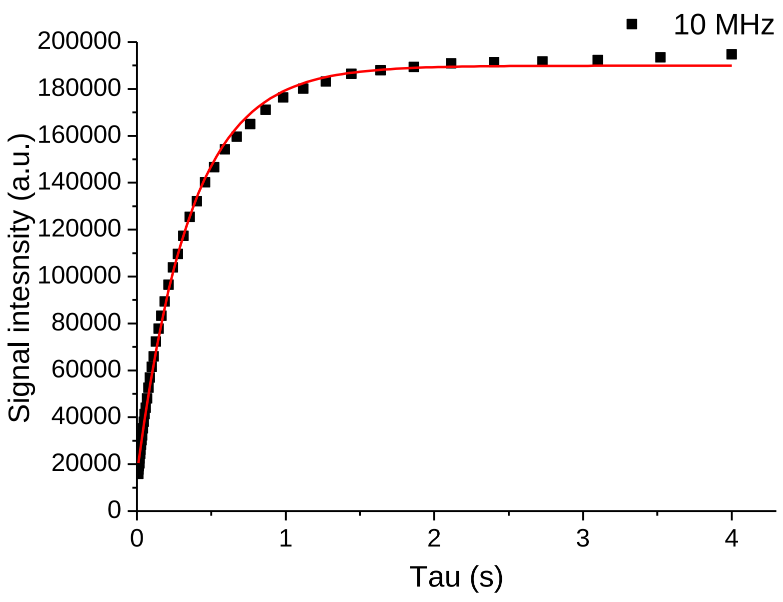

B

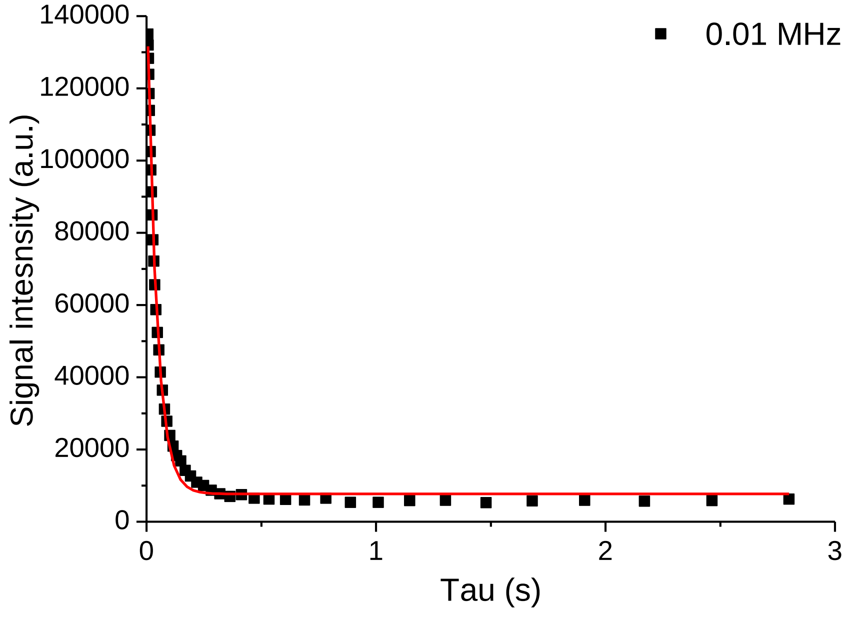

Figure S7. Mono-exponential fit ($M_z=M_0(1-\exp(-t/T_1))$+offset) of the SR of a hind limb muscle, measured at 10 MHz (A, Adj. $R^2 = 0.99844$) and 0.01 MHz (B, Adj. $R^2 = 0.99682$).

A

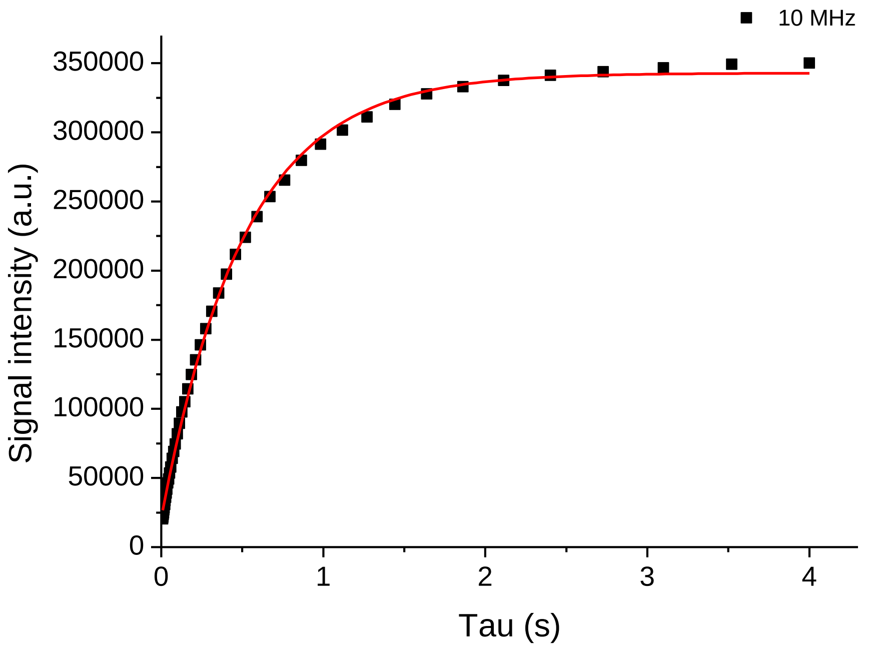

B

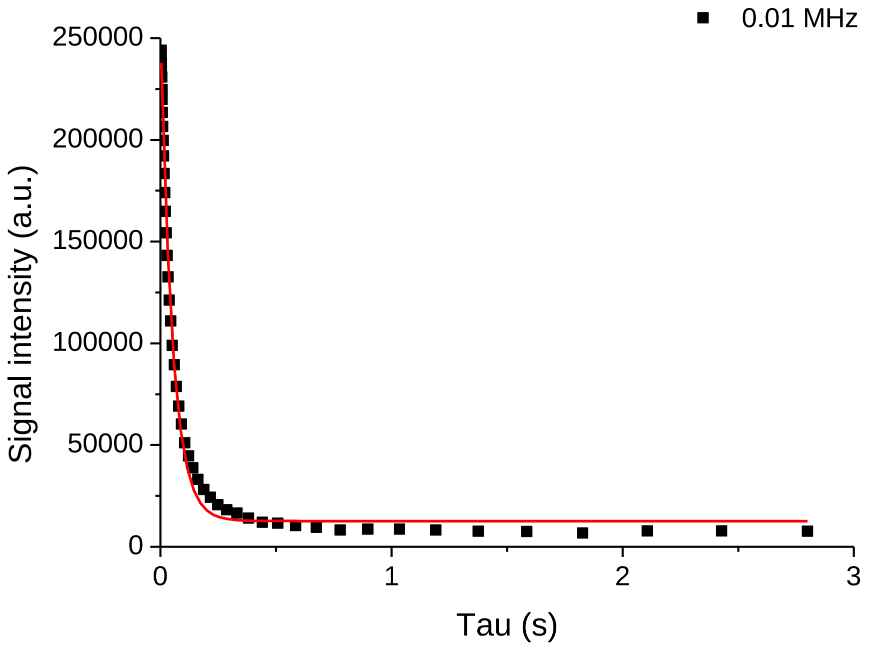

Figure S8. Mono-exponential fit ($M_z=M_0(1-\exp(-t/T_1))$+offset) of the SR data of a TS/A tumour bearing mouse, measured at 10 (A, Adj. R^2= 0.99891) and 0.01 MHz (B, Adj. R^2 = 0.99682).

VI) ***In vitro*** **determination of membrane permeability by relaxometric procedure.**

4T1, 168FARN and TS/A cells, (7, 7 and 5.5 million, respectively), were detached with 0.05% trypsin and 0.02% EDTA in PBS, washed once with PBS and re-suspended in the presence of variable concentrations of Gd-HPDO3A (5-40 mM in PBS). Gd-HPDO3A

(Prohance) was kindly provided by Bracco S.p.A. (Milan, Italy). In order to avoid the internalization of the metal complex, the relaxometric measurements were carried out within 15 min.Then cells were transferred in 5 mm NMR tubes and centrifuged 5 minutes at 0.1 rcf (4°C). Water proton T_1s of the cellular pellet were measured at 0.5 T and 25°C on a Stelar SPINMASTER spectrometer (Stelar, Mede, Italy) by means of the inversion-recovery (IR) pulse sequence with 64 τ increments. A biexponential recovery of the magnetization was observed. The water exchange rates (k_{in} and k_{ex}) across the cell membrane were determined by analysing the IR data in terms of the 2SX model (see ref. 6b in the main manuscript). In the fitting procedure the relaxation rate of the cytosolic compartment (see below), and the GdHPDO3A millimolar relaxivity (4.5 s^{-1} mM^{-1}) were fixed. The relaxation rate values of the cytosolic compartment for each cell line (0.90 s^{-1} for 4T1, 1.10 s^{-1} for TS/A and 1.28 s^{-1} for 168FARN) were determined by suspending similar cell number in the presence of Eu-HPDO3A 50 μM in PBS (synthetized as described in reference [1]) and by fitting the water exchange process fixing to 0.4 s^{-1} the relaxation rate of the extracellular compartment. In these samples the time evolution of the magnetization in the IR experiment was mono-exponential (fast exchange limit) and the relaxation rate values for the two compartments were obtained from the equation:

$$R_1 = R_{1in}V_{in} + R_{1ex}V_{ex}$$

V_{in} and V_{ex} were assessed via the measurement of Gd and Eu concentrations by inductively coupled plasma mass spectrometry (ICP-MS; element-2; Thermo-Finnigan, Rodano (MI), Italy). Since the metal complexes distribute only in V_{ex}, this external volume fraction can be determined by the relation:

$V_{ex} = n^{Gd/Eu}(mol) / (M^{Gd/Eu}(mol/l)*Vol_p(l))$

were $n^{Gd/Eu}$ is the number of Gd or Eu moles determined by ICP-MS; and M is the concentration of the GdHPDO3A and EuHPDO3A solutions added to the cells, and Vol_p is the volume of the wet cellular pellets.
V_{in} is then given by:

$V_{in} = 1-V_{ex}$

VII) Immunofluorescence of GLUT1 transporter and Na^+/K^+ ATPase.

4T1, TS/A and 168FARN were seeded in glass bottom confocal dishes at density of 50000 of cells per well. After 24 h they were fixed with methanol at 4°C for 10 minutes. The cells were permeabilized in 0.1% Triton X-100 in PBS for 10 minutes, saturated in 5% bovine serum albumin (BSA) in PBS for 30 minutes, and incubated with the related primary antibody (Abcam, ab15309 and ab76020 for GLUT-1 and Na+/K+ ATPase, respectively) for 45 minutes at room temperature. Primary antibodies were detected with anti-rabbit Alexa Fluor 488 (Molecular Probes, Invitrogen) used at 1:500 dilution for 30 minutes. Counterstaining was performed with the DNA dye DAPI (Sigma) at 0.5 µg/ml for 5 minutes. Imaging was performed using a Leica TCS SP5-AOBS 5-channel confocal system (Leica Microsystems) equipped with a 405-nm diode, an argon ion, and a 561-nm DPSS laser. Fixed cells were imaged using a HCX PL APO 63x/1.4 NA oil immersion objective at a pixel resolution of 1024x1024.

VIII) Inhibition of GLUT1 transporter and Na^+/K^+ATPase and determination of membrane permeability.

The toxicity of WZB117 ((2-fluoro-6-(m-hydroxybenzoyloxy) phenyl m-hydroxybenzoate), Sigma) and Ouabain (Sigma), inhibitor of GLUT1 and Na+/K+ ATPase, respectively, was assessed by the MTT test (Figure S9 and S10). MTT assay is based on the tetrazolium salts reduction to formazan by mitochondrial succinate de-hydrogenase (SDH), which is quantified spectrophotometrically. The cells were seeded in 96 multiwells at density of 10000 cell/well. After 24 h, WZB117 (0.1 to 50µM) or Ouabain (0.1-1mM) was added to the cell suspensions that were incubated for further 24 h at 37°C (5% CO_2). The medium was then removed and 100 µl of Thiazolyl Blue Tetrazolium Bromide (Sigma) was added at the concentration of 0.45 mg/mL into each well and the plate was incubated for 4 h (37 °C and 5% CO_2). Finally, upon removal of the suspension medium, 150 µl of Dimethyl sulfoxide (DMSO) were added into each well to solubilize the formazan salt crystals produced by the metabolism of live cells. The microplate was incubated at room temperature for 30 minutes and the absorbance was read at 570 nm with iMark microplate reader (Biorad). Cell vitality was reported as percentage of dead cells relative to control. The experiment was performed in triplicate and the data were graphically presented as mean ± SD.
For the determination of membrane permeability, 4T1, TS/A and 168FARN were seeded in 175 cm^2 flask at density of 6 million of cells/flask. After 24 h, cells were incubated with 5 µM of WZB117 and 100 µM of Ouabain respectively, for a further 24 h. The determination of membrane permeability on the treated cells was performed following the same protocol described above.

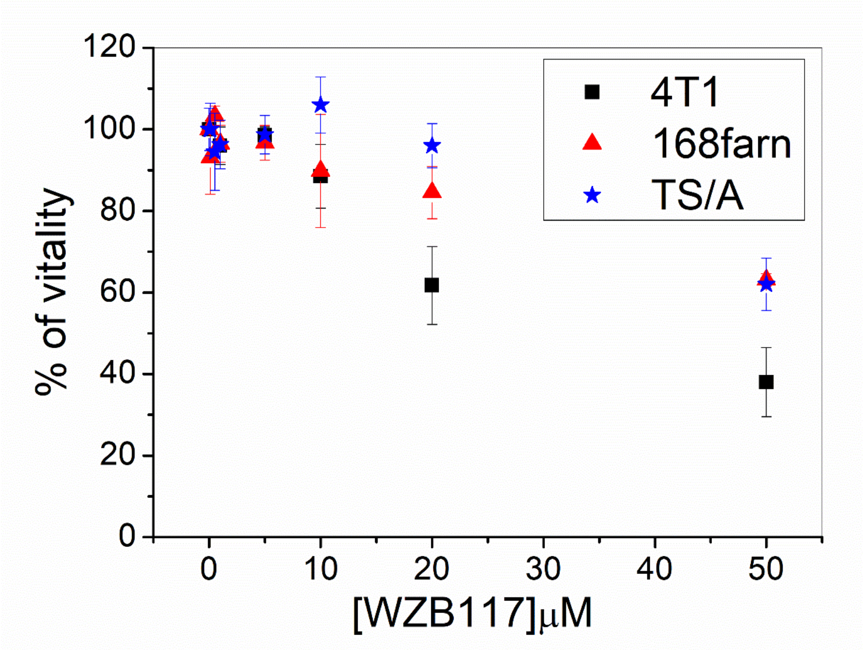

Figure S9. MTT test after inhibition of Glut1: 4T1, TS/A and 168FARN were treated with different concentrations (0.1-50 µM) of WZB117 and after 24 h the vitality was evaluated by MTT test. Cell vitality was reported as percentage of live cells observed in treated samples relative to that observed in control cells. The experiment was performed in triplicate and the data were graphically presented as mean ± SD.

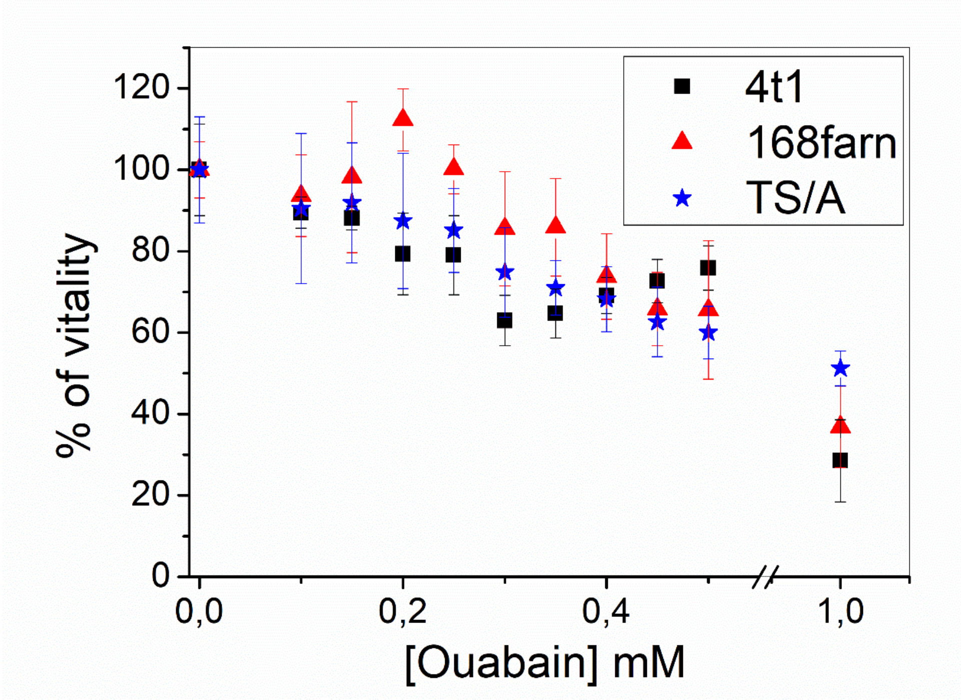

Figure S10. MTT test after inhibition of Na+/K+ATPase**:** The cells were treated with different concentrations (0.1-1mM) of Ouabain and after 24h the vitality was evaluated by MTT test. Cell vitality was reported as percentage of live cells observed in treated samples relative to that observed in control cells. The experiment was performed in triplicate and the data were graphically presented as mean ± SD.

IX) Theoretical Methods: NMRD data analysis.

In the 2SX model (see reference 6c in the main manuscript), the Bloch equations for nuclear magnetic resonance are modified to describe two-compartments (intra and extracellular) in which water exchange modulates the observed relaxation behavior (see Figure 3 in the main manuscript).
The model predicts that the experimental recovery of longitudinal magnetization after 90° pulse (saturation recovery sequence) can be analyzed as a biexponential:

$$M_z = M_0 \cdot \{1 - [(1 - a_s) \cdot exp(-t \cdot R_{1L}) + a_s \cdot exp(-t \cdot R_{1s})]\} \quad [1]$$

where M_z is the instantaneous magnetization, M_0 is its Boltzmann equilibrium value, a_L and R_{1L} are the fraction and rate constant for the apparent component with the longer T_1 ($T_{1L} = R_{1L}^{-1}$), a_S and R_{1S} are the fraction and rate constant for the apparent component with the shorter T_1 ($T_{1S} = R_{1S}^{-1}$), and *t* is the running time for recovery by relaxation. Because a_L and a_S are related ($a_S+a_L=1$), there are only three independent parameters: R_{1L}, R_{1S}, and a_S (or a_L), expressed as:

$$R_{1L} = \frac{1}{2}\left(R_{1ex} + R_{1in} + \tau_{ex}^{-1} + \frac{V_{ex}}{\tau_{ex}(1-V_{ex})}\right) - \frac{1}{2}\left[\left(R_{1ex} - R_{1in} + \tau_{ex}^{-1} - \frac{V_{ex}}{\tau_{ex}(1-V_{ex})}\right)^2 + \frac{4V_{ex}}{\tau_{ex}^2(1-V_{ex})}\right]^{\frac{1}{2}} \quad [2]$$

$$R_{1s} = \frac{1}{2}\left(R_{1ex} + R_{1in} + \tau_{ex}^{-1} + \frac{V_{ex}}{\tau_{ex}(1-V_{ex})}\right) + \frac{1}{2}\left[\left(R_{1ex} - R_{1in} + \tau_{ex}^{-1} - \frac{V_{ex}}{\tau_{ex}(1-V_{ex})}\right)^2 + \frac{4V_{ex}}{\tau_{ex}^2(1-V_{ex})}\right]^{\frac{1}{2}} \quad [3]$$

$$a_s = \frac{1}{2}\left(1 - \frac{(R_{1ex}-R_{1in})(1-2V_{ex}) + \tau_{ex}^{-1} + \frac{V_{ex}}{\tau_{ex}(1-V_{ex})}}{\left[\left(R_{1ex}-R_{1in}+\tau_{ex}^{-1}-\frac{V_{ex}}{\tau_{ex}(1-V_{ex})}\right)^2 + \frac{4V_{ex}}{\tau_{ex}^2(1-V_{ex})}\right]^{\frac{1}{2}}}\right) \quad [4]$$

where R_{1ex} is the extracellular longitudinal relaxation rate (the compartment with longer T_1), R_{1in} is the intracellular longitudinal relaxation rate, τ_{ex} is the external water lifetime and V_{ex} is the extracellular volume fraction (the compartment with shorter T_1). V_{ex} and τ_{ex} are related to V_{in} and τ_{in} values by equilibrium mass balance:

$$\tau_{ex} = \tau_{in}\,(V_{ex}/\,V_{in}) \quad [5]$$

where τ_{in} and V_{in} represent the intracellular lifetime and fraction, respectively.

X) References

[1] S. Geninatti Crich, L. Biancone, V. Cantaluppi, D. Duo`, G. Esposito, S. Russo, G. Camussi, S. Aime, *Magn Reson Med*, **2004**, *51*, 938–944.

Author Contributions

M.R.R. conceived the work, contributed to cell studies (MTT, confocal microscopy, inhibition experiments), animal models set-up and NMRD profiles acquisition. S.B. conceived the work contributed to data analysis and NMRD profiles acquisition. S.P. contributed to cell studies (evaluation of “in cell” water exchange rates, and inhibition experiments). G.F. conceived the design and developed the instrumental setup suitable for the characterization in-vivo of small mice by means of Fast Field Cycling NMR Relaxometry. S.G.C. conceived the work, wrote the paper and contributed to MRI acquisition, ICP-MS measurements and data analysis. S.A. conceived the work and wrote the paper.
